# Oxygen Reduction Reaction in the Field of Water Environment for Application of Nanomaterials

**DOI:** 10.3390/nano10091719

**Published:** 2020-08-30

**Authors:** Rongkui Su, Chuyue Xie, Sikpaam Issaka Alhassan, Shunhong Huang, Runhua Chen, Siyuan Xiang, Zhenxing Wang, Lei Huang

**Affiliations:** 1 School of Environmental Science and Engineering, Central South University of Forestry and Technology, Changsha 410004, China; surongkui@csuft.edu.cn (R.S.); hshunhong@163.com (S.H.); chen12@csuft.edu.cn (R.C.); xsy07231001@163.com (S.X.); 2 School of Metallurgy and Environment, Central South University, Changsha 410083, China; alhassansikpaam@yahoo.com; 3 South China Institute of Environmental Sciences, Ministry of Ecology and Environment of the People’s Republic of China, Guangzhou 510655, China; wangzhenxing@scies.org; 4 School of Environmental Science and Engineering, Guangzhou University, Guangzhou 510006, China

**Keywords:** nanocatalyst, water pollution, oxidation–reduction, hydrolysis

## Abstract

Water pollution has caused the ecosystem to be in a state of imbalance for a long time. It has become a major global ecological and environmental problem today. Solving the potential hidden dangers of pollutants and avoiding unauthorized access to resources has become the necessary condition and important task to ensure the sustainable development of human society. To solve such problems, this review summarizes the research progress of nanomaterials in the field of water aimed at the treatment of water pollution and the development and utilization of new energy. The paper also tries to seek scientific solutions to environmental degradation and to create better living environmental conditions from previously published cutting edge research. The main content in this review article includes four parts: advanced oxidation, catalytic adsorption, hydrogen, and oxygen production. Among a host of other things, this paper also summarizes the various ways by which composite nanomaterials have been combined for enhancing catalytic efficiency, reducing energy consumption, recycling, and ability to expand their scope of application. Hence, this paper provides a clear roadmap on the status, success, problems, and the way forward for future studies.

## 1. Introduction

With the increase in the population of modern society coupled with the rapid development of industry, the anomaly between man and nature continues to surge. This is because the long-term survival of human beings conflicts with the increasingly prominent practical problems such as environmental pollution and scarce Earth resources [1,2]. Man cannot survive without certain resources such as water. When the quality of the available water is compromised, it endangers not only human life but all living creatures. Water is an inseparable element of human beings and therefore any energy devoted towards ensuring that the quality of water is not compromised is worth the price. Over the years, several studies have focused attention on how to efficiently manage water pollution and rationally develop and use water resources, which remains the research and policy direction and the hotspot of today’s global scientific community [3].

In today’s society, water, which accounts for 71% of the Earth’s surface area, has become a source of people’s wanton access to resources. On the contrary, it has also become a victim of human growing demand and social development. Many human activities such as mining, agriculture, manufacturing, and excreta discharge large amounts of pollutants such as organic dyes, heavy metals, oils, and organic chemical reagents into water [4]. Although the existence of these potential pollutants does not necessarily lead to the degradation of the water ecosystem, when the input of pollutants exceeds the capacity of water dilution and assimilation, the deterioration of the water environment and related biological disturbances will inevitably follow, which eventually affects human survival [4]. For example, a survey along the St. Lawrence River found that PCBs polluted the main food source (fish) available for the residents. Among adolescents, the increase in PCB level was associated with the reduction of female sexual maturity, the reduction of male testosterone levels, and the reduction of young people’s thyroid hormone levels. At the same time, the local government had to comply with the health advice to stop eating local fish since it was found to have significant health effects on those who consumed it. This not only had an impact on health and culture but also changed the original food consumption patterns and social interaction patterns [5]. Settlers around this river have deeply recognized the seriousness and enormity of this water pollution. Pollution of the water environment not only limited the basic lifestyle of the people but also curtailed many possibilities for development. It is true that “water is life”; hence, if human beings want to survive on the Earth for a long time, the most effective and cutting edge water pollution control methods must be applied.

**Figure 1:**
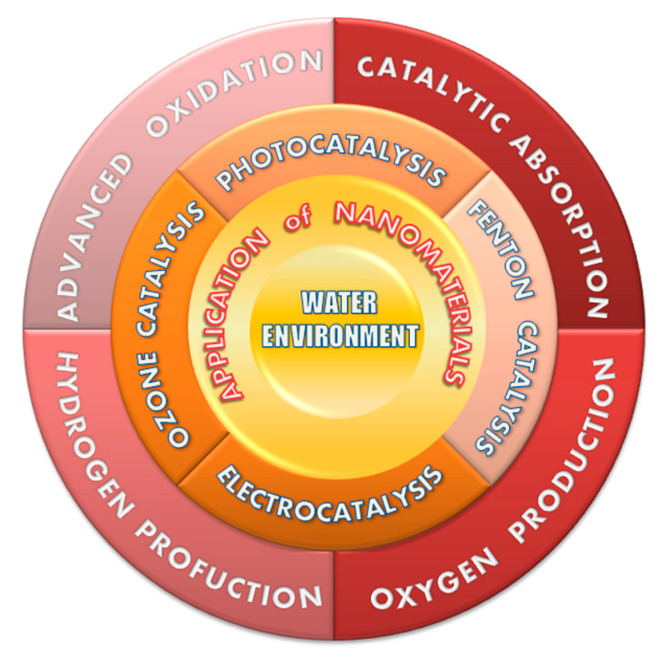
Overview of the application of nanomaterials in the field of water environment.

Furthermore, energy development, utilization, and rapid consumption of fossil fuels have also contributed to serious environmental problems, especially global warming and haze [6,7]. In addition, under the dual pressure of the intensification of the world energy crisis, finding clean and renewable energy alternatives and traditional fossil energy is imperative. As the most abundant element in the universe, hydrogen is three times as hot as oil, and the burning product is water without secondary pollution, which is currently recognized as a high-efficiency energy carrier [2,7,8]. Generally, it forms water or hydrocarbons and nitrogen compounds with oxygen in the natural environment. Since it does not generate greenhouse gases and minimizes secondary pollution, using water resources to produce hydrogen is undoubtedly a better alternative. Therefore, different nanomaterials for the preparation of high-efficiency hydrolysis hydrogen production technology are introduced here, and their application on pollution control and new energy production is described in Figure 1. The use of new catalysts—nanomaterials—and energy such as light and electricity is critical to achieving high efficiency and green catalytic water decomposition. The generation of zero-emission energy hydrogen and oxygen is the best example. Both energy sources are gases, and rapid production capacity and low storage costs have attracted high attention in countries around the world [8,9].

## 2. Classification of Nanomaterial Catalysts in Water

### 2.1. Research Background and Significance of Nanomaterials

To solve the increasing environmental problems and achieve sustainable use of energy, people have shifted their vision from fossil fuels that are prone to global warming and air pollution to new energy sources such as solar energy, water energy, and wind energy [10,11]. At present, how to use these renewable energy sources to achieve high-efficiency, low-energy consumption, and environmentally friendly production capacity or minimize the generation of pollutants has not been achieved. This is attributed to the fact that how to provide catalysts for high catalytic activity, high stability, and sustainable use has not been sufficiently explored [12]. Therefore, to achieve the optimal use of energy and effective management of pollution is the research hotspot of the current society [13].

**Figure 2:**
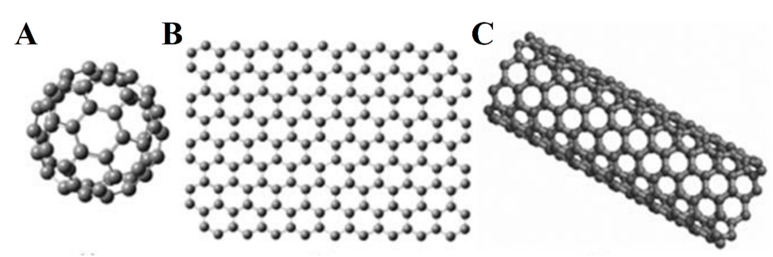
Three different geometrical structures of graphene (GR): (**A**) ball-shaped GR; (**B**) a GR sheet; and (**C**) carbon nanotubes (CNTs). Reproduced [22] with permission from Wiley-VCH, 2016.

Currently, dimensional nanoscale materials in three-dimensional space, henceforth referred to as nanomaterials in this article, have attracted widespread attention in the field of new energy development research and water pollution remediation. The outstanding advantage is that the structural characteristics of the nanomaterials themselves enrich their superior performance, that is, the nanomaterials are different from microparticles and macro objects [2,14]. It is a type of composition or crystal of atoms, molecules, and macro systems. If one direction is at the nanometer level, it is called a two-dimensional nanomaterial, such as nanosheets [15] and nanomembranes [16]; if two directions reach the nanometer level, it is called nanomaterials, such as nanofiber [17,18,19], nanotube [20], and nanorod [21]; and if the three directions reach the nanometer level at the same time, it is called zero-dimensional nanomaterials, such as nanoparticles [22], quantum dots, etc. Three different geometrical structures of GR are shown in Figure 2.

These specific structures allow it to have rich and unique material characteristics, which lay the foundation for high-performance catalytic purposes, such as the existence of small side effects. If the nanoparticles are smaller, the specific surface area is large. The advantage of the huge specific surface area is that it provides more active sites for the reactants and enhances their adsorption capacity [23]. The higher is the atomic ratio of the surface of the particles, the more the surface activity will increase, and the specific surface area will increase accordingly, and the surface tension will also increase at the same time [24]. Ultimately, the outstanding performance of the quantum size effect and the energy gap band of the nanoparticles will become wider and thus have a stronger redox and other properties [25,26]. These microscopic effects endow the nanomaterials with excellent physical and chemical properties, making them more flexible in the field of catalytic reactions.

When nanomaterials are subjected under conditional light exposure, they have a superior structure [7,14,27]. When the electrons in the semiconductor valence band are stimulated to leap forward for the photovoltaic carrier, the valence band that loses the electron will produce a light-borne hole (h^+^) with strong oxidation capability and the conductive belt will have a strong reduction (e^−^). When the electron-empty point migrates to different positions on the surface of the nanomaterial, it will perform oxidative reduction reactions, such as photolysis of hydrogen, water, or oxygen, and the nanocatalyst itself does not participate in the reaction [28].

The high conductivity of nanocatalytic materials can be used. During electrolysis, the electric field action is conducive to the transfer of electrons (e.g., electrolysis in hydropower, electrolysis in water, and electrolysis in water loss), thereby accelerating the response rate [29]. Similarly, the nanomaterials themselves do not undergo any chemical reaction.

As the basis for the realization of the above catalytic reaction, the selection and formation of the catalyst itself are extremely important. To achieve the goal of high-efficiency catalysis, nanocatalysts with strong light absorption capacity or capable of rapid charge separation and migration are needed [10,30]. Generally, the following materials are used to complete high-efficiency redox strategies. (1) Graphene and other nanocarbon related materials: They have a huge surface area. Their huge surface area can not only reduce the total volume as much as possible, but also increase the contact area, reduce the time for the photogenerated electron–hole pair to reach the surface from the inside of the catalyst [31], reduce the probability of recombination of the two, and enhance the catalytic performance. At the same time, its excellent electrical conductivity greatly helps to increase the electron transport rate inside the catalyst. (2) Nano-metal element and compound: Nano-metal element can form new active sites, promote charge transport, and improve light utilization [31,32]; the nano-metal compound can improve redox by preventing electron carrier and hole carrier recombination. The activity can also use the coupling effect with graphene and other materials to further strengthen the stability of the structure and improve the recycling of metal nanomaterials. (3) MOFs and other materials: The high porosity and huge specific surface area of the MOFs provide space for various catalytically active substances, for instance metal oxide clusters and metal groups [12,33]. The use of ligands and metal charge transfer or excitation of metal active sites promotes charge transport. On the other hand, functional groups also help electrons in the separation and transfer of photogenerated carriers, thereby improving photocatalytic efficiency [34].

### 2.2. The Basic Composition of Nanocatalyst

The functions performed by different materials according to the content of the nanocatalyst can be divided into three parts: main catalyst, catalyst, and carrier. The main catalyst component is also called the activation component, and it can have catalytic activity if it exists alone. While the catalyst often exists alone without showing catalytic activity, its addition can make the main catalyst more active [35]. In general, structured catalysts are used to increase the specific surface area of active components or to improve the stability of active structures, such as iron–potassium oxide for ammonia synthesis–alum, oxide–alum, and oxide–oxide alumina catalysts. The modifier’s catalyst can modify the specific activity of the active component, such as the aforementioned iron–potassium, oxide–alum, and oxide catalysts [36]. In the iron contact medium of synthetic ammonia, adding small amounts of aluminum and potassium oxide can increase the catalytic activity of the onboard by 10 times and extend its life [37,38]. At the same time, the carrier is one of the important components of the load-type catalyst and it does not have catalytic activity itself, but it can be used as the skeleton of the catalyst active component [39]. Since nanomaterials have larger and more gaps than their surface area, the active components are fully distributed on the surface of the catalyst, producing so-called active points, and at the same time acting to increase the mechanical strength of the catalyst. Different carriers, catalysts, and preparation methods can have an impact on the catalytic performance of the main catalyst [15].

### 2.3. Nano-Metal Material Monomer and Compounds

#### 2.3.1. Nano-Metal Catalyst

Nano-metal catalyst refers to a nano-level solid catalyst with metal as the main active component, mainly noble metals and transition metal elements such as iron, cobalt, and nickel [40,41]. According to whether the active component is supported on the carrier, it can be divided into unsupported metal catalysts and supported metal catalysts. Unsupported metal catalysts refer to metal catalysts without supports, usually in the form of metal particles. They are also widely used in the construction of nanomaterials because of their strong electrical conductivity, thermal conductivity, and corrosion resistance. According to the composition, it can be divided into single metal and multi-metal [42]. However, due to the easy dissolution of metals into the environment, this type of catalyst will lose catalytically active components [43]. On the other hand, it also increases the possibility of secondary pollution of the environment, which reduces the practicality of this high-efficiency catalyst [32,44]. Based on this, scientists have made a supported metal catalyst. That is a catalyst that supports metal components on nanocarriers [45]. Due to the presence of nanocarriers, the dispersion and thermal stability of metal components are improved, providing multiple voids catalytic site and a certain degree of mechanical strength. Most supported metal catalysts are prepared by impregnating metal salt solutions on support, reducing them after precipitation transformation or thermal decomposition [46]. Therefore, one of the keys to preparing supported metal catalysts is to control the heat treatment and reduction conditions.

Nano-metallic monomers often appear in the form of alloys due to their high reduction activity, large surface area, and unique characteristics of each metal. However, metal nanoparticles are also very easy to oxidize because of their zero valent states. How to continue to maintain the high efficiency and stability of the nano-metallic element in the water environment, recyclability, etc. also requires further exploration [45].

#### 2.3.2. Nanometallic Oxides and Metal Sulfides

At present, it can be seen that, although metal nanoparticles have high catalytic activity, pure metal nanoparticles are prone to flow into the environment, showing weakness in recycling and may cause secondary pollution to the ecological environment [47]. To make full use of metal ions to provide more active bit characteristics, the study of metal oxides and hydroxides has been in progress [14,44].

In the field of catalysis, titanium dioxide is the most widely studied and is most commonly used in combination with other metals because of its good dispersion and tolerance, high-multiplier performance, and cycle stability. Its other good properties include rapid charging performance and higher capacity, embedded lithium reversibility, and good application prospects in the field of lithium batteries [48]. In terms of light catalysis, the excitement of light sources has accelerated the generation of electron-empty pairs with oxidation and reduction capabilities [2,46]. Oxidizing and degradation can be attached to various organic substances such as formaldehyde and some inorganic objects on the surface of the object. Other nano-metal oxides, such as molybdenum dioxide and titanium dioxide, have similar properties, but, because their forbidden bandwidth is only 1.78 eV [29], which can absorb visible light, they have received widespread attention in the field of photoelectric catalysis.

Metal sulfides also exhibit excellent semiconductor catalytic performance. They are similar to metal oxides. The energy band structure is not superimposed, which is a separate energy band, but the additional energy level generated by its holes is close to the valence band [49], making it easier for metal sulfides to accept electrons from the valence band. Taking advantage of this characteristic feature, for example, Sarwar et al. [50] produced a MoS_2_/graphene catalyst by an ultra-fast (60 s) microwave-initiated method. Excellent HER activity and low activation energy of 36.51 kJ·mol^−1^ in the range of 30–120 °C were achieved at the same time. Such composite metal sulfide nanomaterials showed high cycle stability and can be continuously produced under constant potential hydrogen 90 h. The use of oxides and sulfides of such non-precious metals solves the problems of lack of resources and high valent of precious metals such as platinum. It also highlights the commercial value of the Earth’s rich resources such as molybdenum and non-precious metals, and it has both high efficiency and sustainability for industrial production applications through its ideal template.

#### 2.3.3. Nano-Metallic Hydrogen Oxides

Bimetal hydrogen oxides (DHs) are also called laminar hydrogen oxides because of their laminar-like structures. Because of their superior ion exchange performance and interlayer ion storage function, they are widely used in the fields of electrode materials, catalysis, and ion exchange. For example, Teng Wang et al. [15], studied an array of bimetallic hydroxides (LDH) nanometers grown in situ on carbon nanofibers. When they compared it to the original single-metallic hydroxide NiLDH, bimetallic hydroxide NiCuLDH exhibited superior electrochemical performance, including more than 50% higher capacity. The excellent performance is because NiCuLDH had higher conductivity and faster interface charge migration rate. Such composite metal hydrogen oxides provided a clearer and more effective research direction for the effective use of the Earth’s rich materials and the progressive enhancement of electrode performance.

Although nano-metallic oxides have certain adsorption properties, there are particle nuances that are difficult to degrade in water solutions and difficult to recycle. The science, technology, and management of their biosafety are yet to be developed. Compared with this, the use of large amounts of carbon in nature itself reduces the pressure of nature’s decomposition catalysts and reduces the concerns of recycling and degradation. Research on the development of carbon nanomaterials has considerable potential.

#### 2.3.4. Nanocarbon Related Materials (Carbon Nanotubes, Carbon Nano Types, Carbon Nanometers, Carbon Quantum Points)

After British physicists Geim and Novoselov separated single-layer graphite in 2004 by tape stripping, their structural stability and extremely high conductivity have since attracted worldwide attention. From a microscopic perspective, graphite refers to a two-dimensional carbon atomic layer composed of a hexane carbon ring with a hive grid structure [8]. Using molecular orbit theory as the theoretical basis, the hexagonal carbon ring in graphite is because the carbon atom is combined with sp^2^ hybridization to form six carbon–carbon σ keys. Because the σ keys can be high and stable, the graphite plane has good stability. Because every carbon atom in the graphite structure has an orbit that is completely reduced by the flat surface, it can form an π orbit with the adjacent carbon atom. Under such circumstances, the molecular orbit of graphite ole forms a continuous conjugate system, and the π electron can move freely on it, making graphite ole exhibit better conductivity than, e.g., conductive graphite [8,48].

In addition, graphite has a solid and special crystal structure that gives many excellent performances. For example, the advantages of large and abundant porosity increase their chances of contact, conversion, and recycling with reactors [47]. Another example is that they are similar to the crystal structure of the hexagonal honeycomb. The advantage of this structure is that the carrier is not affected by crystal defects in motion or foreign atoms are scattered and have a high load transmission efficiency. This makes the nanocarbon material based on graphite an ideal carrier capable of improving the catalytic activity [28]. These characteristics make the research and application of graphite in the catalytic field quite promising. Through progressive development and research, many other graphite preparation methods have also been produced, such as physical methods: micromachine stripping method, chemical method, extension growth method, gas-phase deposition method, and oxidation–reduction method. The production of these advanced production technologies is not only possible for the widespread use of graphite, but also greatly reduces the cost of preparation of nanomaterials [51].

As a two-dimensional single-layer carbon nanomaterial, graphite is the basic structural unit that forms graphdiyne and carbon nanotubes. They are generally formed by the combination of multilayer carbon atoms with Van der Waals forces and are connected with C-C keys in the carbon atomic layer [52]. There are different structural forms of crystal carbon atoms, such as the formation of zero-dimensional Fuller oleene by envelope or stripping and decomposition to form a zero-dimensional carbon quantum point. The carbon nanotube and thread can be formed by an axial bending method through the graphite belt [28]. In addition, during the formation of a three-dimensional layer graphite ink by the stack method, more than 10 layers of graphite ole can be considered. The different dimensional structures of nanocarbon crystals can provide stronger mechanical strength. If other nanomaterials are loaded, they can achieve the purpose of being highly dispersed and providing a larger microenvironment.

**Figure 3:**
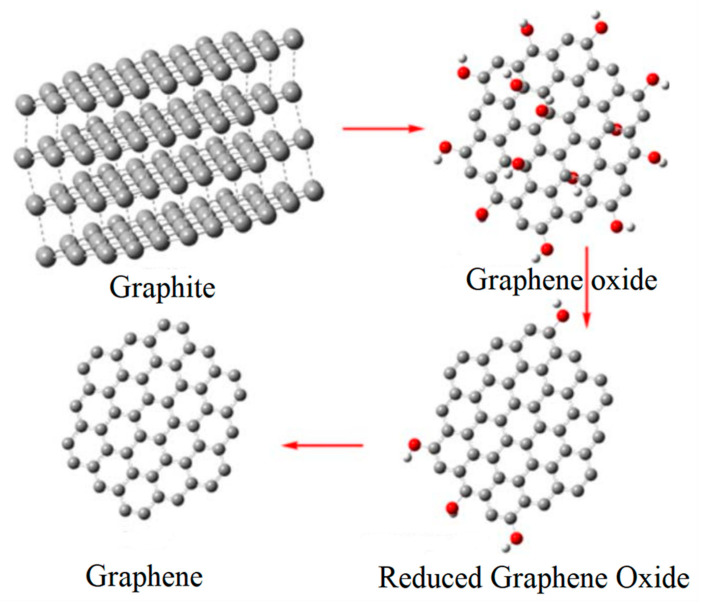
The preparation process of rGO and GO. Reproduced [22] with permission from Wiley-VCH, 2016.

It is more noteworthy that the stacking of graphene sheets due to π–π interaction reduces the catalytic effect. To avoid the occurrence of such phenomena, graphene and its derivatives graphene oxide (GO) and reduced graphene oxide (rGO) exhibit higher electrocatalytic activity and durability [6]. Graphene oxide is a product obtained by the oxidation of graphene. With its rich oxygen-containing functional groups on the surface (–OH, –C=O, –COOH, etc.), it exhibits good hydrophilicity, allowing it to be used in water. It is sufficiently dispersed to play the role in the catalytic degradation of pollutants and often exists as a precursor of self-assembly [49]. At the same time, many studies have shown that the rich functional groups on the surface of graphene oxide can be complex with heavy metal ions or use the interaction of π–π bonds to adsorb organic pollutants. With the help of the hydrogen bonding with their functional groups, they can show an extremely strong adsorption effect. The reduced graphene oxide is prepared based on graphene oxide. Due to the loss of related oxidation groups, it exhibits relative chemical stability. The preparation process of rGO and GO is shown in Figure 3. The experimental results of Alammari et al. [16] showed that depositing different Pt/Ru on reduced graphene oxide generates extremely high catalytic activity for ORR, and its governance and the application of green energy are very promising catalytic materials.

### 2.4. Nanometallic Organic Skeleton MOFs and Other Materials

Nanomaterials such as transitional metal oxide and titanium dioxide, which have been widely developed and studied have become the most widely used semiconductor catalytic material at present due to their appropriate oxidation–reduction power, low cost, and high stability [53]. However, two problems limit the improvement of catalytic efficiency: a larger gap makes them insufficient to absorb sunlight efficiently, and, although the particle size is reduced to a nanometer, it can increase its surface area to a certain extent, but there is a strong reunion between the particles [54]. At present, to solve such problems, the metal active component should be mixed with porous material crystalline aluminum silicate (boiling stone), rich orifice, and hole rich in nano or sub-nano size. However, the impervious light of the boiling stone itself does not absorb visible light and ultraviolet light [54], which affects the catalytic efficiency of such composite porous materials. Therefore, from the perspective of porous materials, metal–organic frames (MOFs) not only make up for the lack of boiling and boiling stone types but also introduce exposed metal centers. This allows them to interact directly with metals, showing that the pore material is in the catalytic field warranting a more excellent performance [55].

MOFs are a three-dimensional cyclical network structure consisting of metal ions, metal clusters, and organic compounds [56]. Their narrower energy gap zone enhances the absorption capacity of nanocatalysts to solar light and improves the efficiency of light utilization. In addition, the central ion and the adjustability of the fitting structure grant the catalytic rate greater room for improvement. Moreover, as with the high gap characteristics of the boiling stone, the highly even distribution of metal active points promotes the catalytic reaction. Additionally, the tiny pore structure also improves the accessibility of each active point, while the highly developed large aperture channel serves as a channel for the transportation of reactants.

Current research results show that pure MOFs medium luminous electron empty cave is extremely complex, which affects light catalytic activity. Therefore, to delay the composite of the photovoltaic electron-empty cavity so that it no longer limits the catalytic efficiency, it is necessary to load nanoparticles on the MOFs medium and cover the nanoparticle surface with organic molecules or to modify the surface. For example, Dengrong Sun et al. [54] prepared and studied the metal–organic frames (MOFs) containing Zr and Ti. Their results confirm the strategies of multiple components needed to improve light absorption and promote ionization providing a multi-functional light catalytic process ideal platform.

More notably, most MOFs are unstable in water and may decompose in a high oxidizing environment, thereby limiting their high performance. Therefore, future research should propose new strategies for this, such as transforming MOFs or equipping them (e.g., MOFs@TiO_2_). At the same time, the pyrolysis conversion mechanism of MOFs has not been clearly defined, and the problem of controlling fine structures remains to be solved, which is essential for the catalytic performance research of MOFs and their derivatives. Therefore, it is also necessary to conduct a step-by-step study.

### 2.5. Research Problems and Directions of Nanomaterials

Although the research on nanoscale materials has already started and made some progress, there are still some common problems that need to be resolved going forward: (1) Most of the nanomaterials currently developed have large bandgap widths. This structural limitation has led to the fact that it absorbs shorter-wavelength ultraviolet light in sunlight, but most of the visible lights in sunlight are not fully utilized. This results in low light utilization, and, even if a photocatalyst with a narrow bandgap were selected, it would face an insurmountable overpotential problem. In addition, the low light absorption rate will affect the effective progress of photocatalysis. (2) The reduction of the size of the metal nanomaterials increases the specific surface area of the nanomaterials, but, due to the strong molecular force between the metal particles, the metal nanoparticles easily agglomerate to form secondary particles. This is not conducive to providing active sites and greatly affects the stability of the reaction. (3) The photo-generated electron–hole pairs generated by excitation under light irradiation are also prone to recombination during the process of migration to the catalyst surface, and the lifetime is not high, which is also a major reason for affecting the catalytic efficiency.

To solve these problems, scholars have found that loading functional metal materials on the nanocarbon carrier through surface modification or modification (doping, deposition, photosensitization) and other methods can combine the advantages of the two and perform in the field of catalysis. Shatila Sarwar et al. [50] used pure molybdenum disulfide, pure graphene, a physical mixture of molybdenum disulfide + graphene, pure Pt, and molybdenum disulfide/graphene composite materials for electrolysis of water. It was found that, at the same current density under the condition of 10 A/cm^2^, the overpotential of the molybdenum disulfide/graphene composite material is as high as 183 V, which is more than three times that of pure Pt and the physical mixture of pure molybdenum disulfide, and molybdenum disulfide + graphene is very catalytically active and low. Every pure graphene supported by molybdenum disulfide shows catalytic activity. From this, we can draw some conclusion that, comparatively, a multifunctional composite nanocatalyst can utilize the synergy between nanometal particles and graphene nanocarbon materials to provide more active sites for the reaction than a single catalytic material. This has a higher catalytic performance. In this way, the reasonable combination of strengths and weaknesses in actual research is also the main research method and direction of the current nanomaterials research community.

## 3. Advanced Oxidation

### 3.1. Advanced Oxidation Research Status and Content

With the rise and continuous high-speed development of industry and agriculture, a large amount of artificially synthesized organic waste liquid directly or indirectly flows into the environment, such as persistent organic matter, dye-containing wastewater, oilfield wastewater, and high-concentration permeable liquid. Organic pollutants can migrate long distances and exist in the environment for a long time, hence causing serious harm to human health and the environment. Therefore, it is necessary to roll out cutting edge studies on how to effectively and efficiently remediate organic wastewater that is difficult to degrade.

Advanced oxidation technology (AOPs) is the use of light or electricity to accelerate the electron transfer rate on the catalyst under certain conditions. It also promotes the activation of the oxidant by the catalyst to produce active radicals (•OH, SO_4_^−^•, etc.) to oxidize and degrade organic pollutants. Ultimately, AOPs becomes a chemical technology that is harmless to the environment or that pollutes smaller molecules. Compared with the biological method and physical method for treating pollutants in water, the advanced oxidation method has a greater advantage due to the following reasons. (1) Fast reaction rate and mild reaction conditions: Under normal temperature and pressure conditions, high rate oxidation remediate pollutants in water. (2) Strong oxidation ability and low selectivity: The generated active radicals (•OH, SO_4_^−^•, etc.) have a higher oxidation potential than general oxidants and can destroy most organic macromolecules. The conjugated structure makes it a non-toxic or low-toxic small molecule. (3) Small secondary pollution: Catalyzing hydrogen peroxide and sulfate under the conditions of light or electricity to achieve the complete mineralization of organic pollutants, the risk of secondary pollution can be minimized.

The use of an advanced oxidation reaction to degrade organic pollutants is based on external energy (photovoltaic energy) and oxidant or water, which can be divided into direct oxidation methods and indirect oxidation method. The direct oxidation method achieves the oxidation degradation of pollutants by using active free radicals • OH or SO_4_^−^• produced by oxidants, such as photolysis oxidation, or direct oxidation of target pollutants on the surface of the anode, such as the direct oxidation method of the anode. The indirect oxidation method, in general, under the presence of a catalyst, promotes biological free radicals (ozone and hydrogen peroxide), water produced by the oxidant (ozone and hydrogen peroxide), or water. When this happens, it decomposes various organic matter pollutants, such as photocatalytic oxidation method and cathode indirect electrolysis method. The indirect oxidation method is different according to the phase of the catalyst and can be divided into equal catalytic oxidation and non-equivalent catalytic oxidation. The all-phase catalytic oxidation refers to metal ions as active components to accelerate the life free radicals of oxide production, the process of contaminants in the oxidized degradation water, such as the average Fenton oxidation method, Fenton oxidation method, the equivalent ozone oxidation method, etc. However, because the phase of the homogeneous reaction catalyst is the same as the target liquid, it will easily lose active ingredients, and it is difficult to recover. The influx of metal ions into the environment may also cause secondary pollution, which significantly limits its scope of use. In heterogeneous catalysis such as oxidation, insoluble solids such as metal oxides or metal hydroxides are used to accelerate the transfer of electrons in semiconductor materials, such as heterogeneous Fenton oxidation and heterogeneous ozone oxidation unlike in metal ions. It is no longer restricted by the acidity and alkalinity of the medium. It has been widely concerned and deeply studied by the research community.

The derivative technology of advanced oxidation reaction depends on highly lively and non-selective substances [57], which mainly contains two types: (1)•OH; and (2)SO_4_^−^•.

(1) •OH: It is generally produced by the reduction of hydrogen peroxide by metals, and the by-product in the acid medium (pH ≈ 3) is non-toxic H_2_O, which shows the excellent characteristics of environmental friendliness. The high oxidation potential after fluorine is shown in Table 1. Non-selective oxidation can be achieved quickly, not only to degrade most organic pollutants such as allylins and phenols into small molecular carbon dioxide and water that are harmful to the environment, but also for the treatment of heavy metals and the disinfection of microorganisms. This leads to a large degree of reduction in the burden of the environment, thus it has attracted much attention as an environmentally friendly oxidizing material.

Fe^2+^ + H_2_O_2_ + H^+^ → Fe^3+^ + HO• + H_2_O (1)

Although activated free radical OH is widely used in organic wastewater treatment due to strong oxidation and non-toxic products, it is also subject to qualitative limitations, that is, the most suitable pH is about 2.8–3. However, the pH value depends on the composition of the wastewater, which will greatly limit the widespread application of active free radical •OH in water pollution management [57]. Another aspect in terms of non-selective oxidizing pollutants means that the number of oxidizing objects increases and the utilization rate of hydrogen peroxide itself cannot reach a very high level. When this happens, it results in a large amount of hydrogen peroxide waste in its oxidant form. To solve the above problems, the development of a non-equivalent oxidation method or the development of a new active free radical-step-release •OH-related binding is needed.

(2) SO_4_^−^•: Active free radical SO_4_^−^• is produced by the activation of sulfate (S_2_O_8_^2-^) through ultraviolet light, high temperature, or transitional metals. There is a high oxidation activity similar to OH, as shown in Table 1. Compared with the limitation that the hydroxyl group can achieve strong oxidation under acidic conditions, the scope of SO_4_^−^• with pH (pH = 2–8) is more extensive, and it can still have high oxidation under alkaline and neutral conditions of the acid environment activity. Additionally, the stability of SO_4_^−^• and the selectivity of oxidation objects make it quite promising in organic wastewater treatment.

S_2_O_8_^2−^ + M_2_ → M_3_^+^ + SO_4_^−^• + SO_4_^2−^ (2)

Most of the sulfate that can produce SO_4_^−^• active free radicals are trans-sulfate, such as sodium sulfate and potassium sulfate, and per-sulfate is PMS. The key to advanced oxidation reaction technology is how to efficiently activate sulfate to produce SO_4_^−^•. A variety of transitional metal ions (Fe(II), Cu(II), Co(II)) can reduce the asymmetric structure of peripheral sulfate activating the domestic free radical SO_4_^−^• to achieve better response to the oxidation of organic pollutants. However, the problems of the high toxicity of heavy metal ions in the phase system, high cost of preparation, large dissolution, and poor stability limit its widespread application in activated sulfate, making the advancement of efficient multiphase catalysts more effective.

Based on the active free radical oxidation mechanism, other acting mechanisms make the catalyst exhibit better oxidation degradation performance. It is the interface effect of the solid-phase catalyst, which can adsorb the pollutants, and then use the combined reaction of the catalyst ion and the organic matter to form a complex. This can lower the reaction activation energy and thus facilitate the oxidation of the oxidant. The second is that the synergy of the catalytic material itself can activate active bit points such as metal ions to produce more active bit points, and maintain better catalytic oxidation performance due to the larger porosity and surface area.

Among the above three catalytic oxidation reaction mechanisms, active free radicals (•OH and SO_4_^−^•) have the characteristics of extremely high redox potential, high-efficiency oxidation, and the ability to thoroughly mineralize most pollutants. In terms of the specific technical application of the advanced oxidation method, it can be divided into photochemical oxidation and electrochemical oxidation according to different energy sources; according to the type of oxidant used, there is currently Fenton oxidation (Fenton-like oxidation) and ozone oxidation.

### 3.2. Photochemical Oxidation

In general, the light catalyst carrier must be activated by light before it has a light catalytic effect. Improving its material organizational structure (increased porosity, surface area, etc.) is the prerequisite basis for improving light catalysis. Therefore, a good light catalyst carrier should generally have the following characteristics: good luminosity and suitable meter area; strong adsorption of degraded pollutants or target oxides; active components have a better binding force without limiting the catalytic activity; easy solid–liquid separation conducive to the transmission between different phase states; and chemical stability that can exist for a long time.

The use of photochemical oxidation to degrade organic pollutants can be mainly divided into direct photolysis oxidation method, indirect photolysis catalytic method, and comprehensive photooxidation method (photo Finton oxidation method, photovoltaic oxidation method, etc.). The direct photolysis oxidation method refers to the decomposition of water to produce aerobic activity •OH to directly oxidize and degrade organic matter under the conditions of light. However, in this case, the rate of domestic free radicals is slower; thus, when using photochemical oxidation, more consideration should be given to using photocatalysts to improve the oxidation efficiency. The indirect photooxidation reaction is also called the photocatalytic oxidation method, which mainly absorbs the photomagnetic radiation of a specific wavelength. It stimulates the separation of light borne fluids produced on the surface of nanomaterials. The isolated phosgene flower has strong reduction oxidation and promotes the rapid production of decomposing water •OH to destroy the stable conjugate structure of organic pollutants. This method can be used to decompose substances that are difficult to degrade such as chlorinated fat hydrocarbons and chlorinated aromatic hydrocarbons, pesticides, and dyes contained in wastewater, as well as wastewater containing cyanide, chrome ions (Cr), and heavy metal ions.

In general, the light catalytic realization has a wide range of applications for the degradation of pollutants, that is, it can be achieved with sunlight. However, some factors limit the use of visible light, that is, most nanomaterials are prohibited from bandwidth and can only absorb the ultraviolet light of short-wave radiation. To be able to degrade pollutants more efficiently, future research should design more effective light catalysts. It is usually the surface modification or modification of the original nanocatalytic material that improves the use of sunlight.

For example, Soo et al. [17] adjusted the synergistic morphology and particle crystallinity to achieve the best photocatalytic performance of electro spun TiO_2_ nanofibers, which provided new insights for improving light utilization. In this study, they demonstrated that the synergistic combination of low fiber diameter, high crystallite size, and anatase/rutile mixing ratio achieved the best methylene blue degradation rate constant (0.04100 min^−1^). Optimized with the help of a response surface, according to the electrospinning parameters of the obtained fiber diameter response, it is known that the crystallinity in TiO_2_ particles is a key determining factor in the material. The crystallization form of TiO_2_ usually exists in three main phases: anatase, rutile, and brookite. Anatase TiO_2_ crystal has the highest photoactivity among the three crystal phases, mainly because it has an indirect bandgap in the crystal, thereby prolonging the separation time of electron–hole pairs, allowing more freedom of oxidation type base release.

Starting from the goal of improving the light source, Navarro et al. [59] tested the degradation efficiency of amalgam lamps to azo dyes. The photometric method showed that the amalgam lamp system can provide high incident photon irradiance (6.30 × 10^−5^ mol/cm^2^·s). In addition, the amalgam lamp derived AOP can decolorize the dye with a pseudo-first-order reaction of 0.654–4.0081/min and decolorize the dye at low dye concentration, low pH, and high H_2_O_2_ concentration until it reaches the maximum value. It can be stated that the amalgam lamp can be an ideal choice for AOP rapid degradation dye replacement light source.

### 3.3. Electrochemical Oxidation

The advanced electrochemical oxidation method refers to the use of electric fields to accelerate the transfer rate of electrons without the use of chemical oxidants. Directly, it promotes the oxidation of the reaction in the anode or produces strong oxidizing substances in the cathode (e.g., •OH). Indirectly, it promotes the reaction on a high-efficiency catalytic oxidation method. Among them, the synergy of the use of strong oxidative active free radicals in the cathode electrocatalytic oxidation method can increase the conversion rate of the domestic free radicals of oxide products such as water. Alternatively, it can be used for cleaning, pollution control, and significant high-efficiency catalytic performance in organic wastewater treatment.

**Figure 4:**
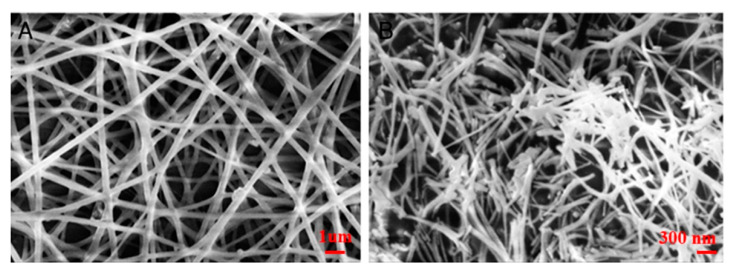
The SEM image (10% load) of the PNZ: as-spun fiber (**A**); and the calcined fiber (**B**). Reproduced [60] with permission from Elsevier, 2014.

**Figure 5:**
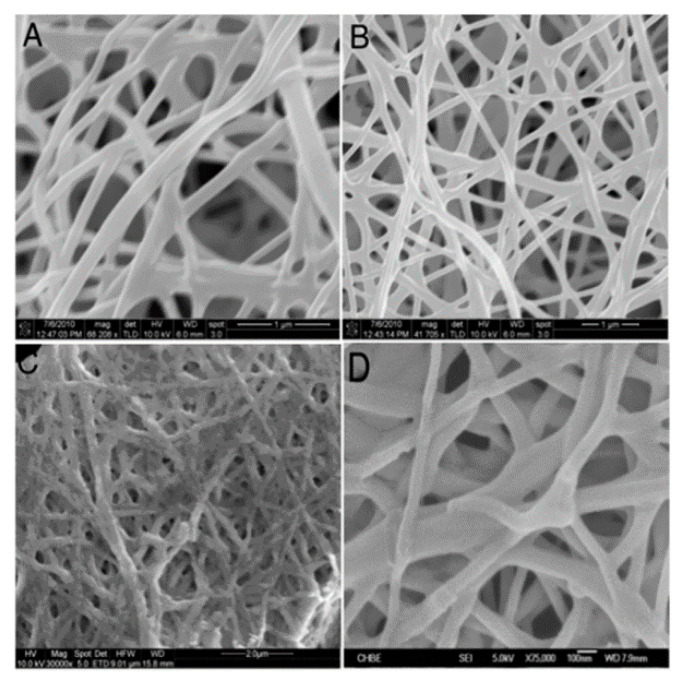
The SEM images of PNZ: as-spun fibers (50% loading) (**A**,**B**); and calcined fibers (**C**,**D**). Reproduced [60] with permission from Elsevier, 2014.

In the electrocatalytic oxidation system, the development of electrode materials with high destructive performance is the core of catalytic electrolytic organic pollutants. This requires not only the stability and corrosion of electrode materials in the water environment but also the high overpower and excellent reduction activity in the pursuit of high performance. At present, because of this problem, better research is to use electrospinning technology to make nanofibers. Because of its simplicity, low cost, and ease of use, it is the most popular technology in the production of functional nano- and ultrafine fibers. Sundarrajan et al. [60] also used electrospinning technology to prepare one-dimensional inorganic–organic composite nanofibers in a solution containing polyvinyl alcohol (PVA) and an appropriate amount of nickel and zirconium ion aqueous precursors, indicating that the addition of precursors would lead to the conductivity of the polymer solution increases. After calcination, PVA/nickel acetate/zirconia (PNZ) fibers retain the original morphological characteristics of as-spun nanofibers. It is prepared according to different precursor loading weight (5–50%) percentage. The average fiber diameters before and after calcination are shown in Table 2. When adding 10 and 20 wt% precursor, the fiber morphology did not change significantly, but a slight decrease in fiber diameter was observed. When the precursor concentration was further increased to 50%, a further decrease in fiber diameter was observed (Figure 4 and Figure 5). X-ray diffraction is used to identify the crystal properties and analytical tools of the final product to clarify the formation path of the ceramic phase and the systematic evolution of the morphological characteristics of the as-spun and calcined fibers. Nanofibers can be used in the biomedical field.

Palaniswamy Suresh Kumar et al. [18] also used a simple and scalable electrospinning technology to prepare one-dimensional TiNb_2_O_7_, inserting lithium to form a performance configuration (Li/TiNb_2_O_7_) and found that the reversible intercalation of lithium (3.45 mol) with good capacity retention characteristics. Li-HEC optimizes electrochemical performance based on TiNb_2_O_7_ anode and AC counter electrode in non-aqueous medium. Li-HEC delivers very high energy and power densities, about 43 Wh kg^−1^ and 3 kW kg^−1^, respectively. In addition, the activated carbon AC/TiNb_2_O_7_ Li-HEC derived from coconut shell can be recycled 3000 times, and about 84% of it maintains the initial performance. Therefore, in principle, lithium-ion hybrid electrochemical capacitors (Li-HEC) are expected to meet the necessary needs, and shortly will become a high-energy and high-power energy storage device for power supply and be used in automobiles and other fields.

Jing Wang et al. [39] studied the synthesis of manganese copper sulfide (MCS) through hydrothermal synthesis. They shown that the significant increase in electrochemical conductivity enables MCS nanocomposites to have improved electrocatalytic kinetics. This method will contribute to a cheap and convenient synthesis strategy for transition metal-based nanostructures for functional applications in the electrochemical and catalysis fields. Furthermore, electrocatalytic degradation can handle heavy metals. Nidheesh et al. [21] conducted an anode direct oxidation experiment on anode oxidizing arsenic in two different electrode systems (Pt/Ti-graphite and Pt/Ti-graphite felt). Under the same current conditions, electrolysis was 60 min. For Pt/Ti-graphite, the removal rate of arsenic was 60%. However, the Pt/Ti-graphite felt system showed that As(III) was completely oxidized to As(V) within 10 min of electrolysis. The oxidation of arsenic is mainly due to the attack of hydroxyl radicals formed by the oxidation of water on the surface of the anode. The anode oxidation process that uses Pt/Ti anode for pollutant oxidation is also a mature technology for direct oxidation of different pollutants.

It can be said that, although the rate of oxidation and degradation of organic wastewater is increased through electrocatalysis, for efficient catalysis, high power consumption is an inescapable problem of the electrochemical reaction. To this end, chemical oxidants (e.g., hydrogen peroxide and ozone) are used to optimize catalytic oxidation and the electro-absorption effects have shown advantages such as easy control of the response, mild conditions, and a significant increase in degradation rate. It provides better prospects for the future development of electrochemical oxidation.

### 3.4. Fenton Oxidation

In general, the Fenton oxidation method refers to a method of oxidizing an organic compound to an inorganic state by using a strong oxidizing hydroxyl group after mixing ferric iron and hydrogen peroxide under acidic conditions (pH 2–5). Because it can reach the oxidation potential second only to fluorine without the supply of external energy, it has become a hotspot in advanced oxidation technology research. With the in-depth study and continuous optimization of Fenton reaction in the past hundred years, using the synergistic effect of external strengthening conditions, optical Fenton [61], electric Fenton, microwave Fenton, ultrasonic Fenton, and other comprehensive technologies have been derived. These comprehensive research technologies do make full use of ferrous iron and hydrogen peroxide improves the efficiency of hydroxyl formation, and achieve the purpose of promoting Fenton oxidation reaction. However, to use Fenton oxidation reaction in organic wastewater to achieve efficient oxidative degradation of organic matter in water for water pollution control, it may be subject to several limitations: (1) The traditional Fenton reaction itself has high requirements for pH, and the best effect can only occur under moderately acidic conditions. As the pH increases, it will form iron precipitate. (2) According to the organic load, high temperature will cause the decomposition of oxidant hydrogen peroxide, which may consume more reagents, resulting in higher operating costs. (3) Although hydrogen peroxide is non-toxic, too much investment will cause the original ferric iron reagent to prematurely oxidized to ferric iron, which reduces the reaction efficiency. (4) The catalyst after the reaction needs to be separated.

To break through the limitations of Fenton’s reagents, heterogeneously catalyzed Fenton-like reactions have shown sufficient advantages. The reason is not only by fixing transition metal ions on the surface of nanomaterials but also solving the problem of transition metal ions flowing into the environment. The far-reaching effect also accelerates the reaction rate through the coupling of nanocatalytic materials. For example, Jiang Li et al. [62] modified in-situ LaFeO_3_ with inert La_2_O_3_ oxide by one-pot synthesis, which improved some important surface properties, such as surface defects, H_2_O_2_ adsorption capacity, Fe^2+^ concentration, charge transfer rate, and resistance to iron leaching. In the performance evaluation, the La_1.15_FeO_3_ (L_1.15_FO) composite material showed the highest Fenton activity (0.0402 min^−1^) among many materials. Taking activated H_2_O_2_ for oxidizing methyl orange as an example, it is 2.5 times the original LaFeO_3._ It is worth noting that the in-situ electron paramagnetic resonance analysis and free radical scavenging test revealed the rapid generation of single-line oxygen of the main reactive species on L_1.15_FO and proposed a novel non-free radical activation mechanism going forward. This improved performance is attributed to the strong coupling effect of nano-LaFeO_3_ and -La_2_O_3_ in the hybrid, which fine-tuned the LaFeO_3_ perovskite, indicating the excellent surface performance of Fenton catalyst.

At the same time, to further solve the problem of catalyst separation, some studies have tried to fix iron on a large volume to make it easy to separate the matrix (e.g., zeolite and MOFs.) for iron exchange and transfer, which can reduce the separation trouble in the later catalyst treatment process. Alternatively, a magnetic force can be used to separate the iron from the wastewater. Such a Fenton catalyst can be prepared by co-precipitating Fe_3_O_4_ on rhombohedral clay. This series of solutions provides bright development prospects for Fenton-like oxidation and degradation of organic wastewater.

### 3.5. Ozone Oxidation

Ozone is also called triatomic oxygen, which is carried by oxygen molecules. When oxygen atom reacts with ·OH, the rest will be combined into the product that oxygen enters a stable state and can be produced at a normal temperature. For ·OH and ·O (Equation (3)), with high oxidation potential, the effect is significant in the deodorization, decolorization, and removal of pollutants of sewage, and the by-product is non-toxic and harmless to oxygen. It can be seen that ozone serves as an efficient oxidant to achieve no secondary pollution.

O_3_ → O_2_ +·O, ·O + H_2_O →·OH (3)

It is currently believed that there are two ways to respond to ozone and organic matter. The first reaction is the direct reaction of ozone in the form of oxygen molecules with organic matter in water bodies. However, this method is more selective and has a better oxidation degradation effect on the organic matter with double keys, such as aromatic hydrocarbons and unsaturated adipose hydrocarbon organic compounds. The second way is to rely on alkaline conditions for ozone to decompose into a water body to produce an active free hydroxyl, which has an oxidizing reaction with organic compounds (Equation (4)). In addition, in recent years, to improve the efficiency of oxidation and degradation, technologies such as UV/O_3_ and H_2_O_2_/O_3_ have been developed over time (Equation (5)). These combined technologies are more efficient than pure ozone oxidation technologies. It is worth noting that the key process parameter in H_2_O_2_/O_3_ technology is the O_3_/H_2_O_2_ concentration ratio. At higher values, the process tends to become ordinary ozonation, and, at lower values, the removal effect of H_2_O_2_ on HO• may occur: H_2_O_2_ + HO• → HO_2_• + H_2_O. At present, laboratory-scale peroxide treatment has proved to be very effective for the degradation of micro-pollutants [57].

O_3_ + H_2_O→ (OH-)HO + 1/2O_2_ + HO_2_ (4)

O_3_ + hv → O_2_ + ·O or H_2_O_2_ + 2O_3_ → 2HO• + 3O_2_ (5)

There are two main mechanisms in the phase-catalyzed ozone oxidation system: (1) the addition of metal ions can promote the biological free base of ozone molecules; and (2) the combination of metal catalysts and target reactions forms susceptibility to ozone degraded complex. To solve the problem of unrecoverable and metal pollution common to homogeneous catalysts, a lot of research has focused on the technology of ozone oxidation of non-uniform catalysts. Not only can it efficiently catalyze the generation of hydroxyl radicals, but it can also reduce the risk of secondary pollution and is very popular as a catalytic material that is convenient for recycling. The Ti-Co @γ-Al_2_O_3_ compound catalyst prepared by Wenquan Sun et al. [63] used impregnation and soluble-gel to degrade biochemical tailings from the coal chemical industry and achieved impressive results. When biochemical tailings were used to catalyze ozone oxidation degradation experiments, the results show that the best conditions for degradation performance were: reaction time is 30 min, pH is 8.2. ozone flux is 30 mg/min, and catalyst usage is 20 g/L. In the catalytic system, the total phenol and total organic carbon removal rates of biochemical tailings are 66.1% and 57.6%. By adding tert-butyl to the catalytic ozone oxidation system, the mechanism of catalytic ozone oxide degradation organic pollutants was studied. The decline in chemical oxygen demand in biochemical tailings was mainly due to the synergy between Ti-Co@γ-Al.

## 4. Adsorption Catalysis

### 4.1. Heavy Metal Wastewater Treatment Status and Solutions

Heavy metal pollution has become a serious environmental problem globally, mainly from industrial wastewater, domestic sewage, and atmospheric input. In this process, most heavy metals eventually flow into the water, and the ecological environmental problems caused have caused great concern to governments, development agencies, and researchers. Ficken et al. [64] showed that the abundance of species within the survey area was negatively related to the sediment concentration of six heavy metals (copper, nickel, lead, zinc, cadmium, and mercury). In addition, the same study reported that the rapid decline of amphibians and fish and other freshwater vertebrate populations generally implies that freshwater systems were contaminated by heavy metals from industrial and agricultural sources. The effects of these highly toxic metals on the human body not only come from the drinking of heavy metal polluted water but also stems from the fact that humans rely heavily on aquatic food sources such as fish, turtles, and other amphibians. In the long run, it affects and disrupts normal metabolism in the human body, and in a worse scenario case, death may occur. Therefore, finding the technology for efficient treatment of heavy metal wastewater is currently the most urgent problem in the field of wastewater treatment.

Common heavy metal treatment methods mainly include chemical precipitation, biological treatment, membrane separation, adsorption, capacitive deionization (CDI), and electrodialysis. However, their current research level is insufficient, which has delayed the development of heavy metal wastewater treatment. For instance, in chemical precipitation, although it can cause metal ions to precipitate, the product from these precipitates cannot be recycled, which ultimately leaves a deleterious effect on the environment upon which it is disposed of. The biological treatment method has obvious successes and strong universality, but there are still many problems to be solved at the current technological level. The membrane separation method, on the other hand, has the advantage of a high purification level [65]. However, the production cost of changing the membrane is high and it is susceptible to oxidation. To solve these series of problems, the adsorption catalysis method of nanomaterials provides a relatively wide application space in the field of heavy metal wastewater treatment. Because of its high efficiency, low cost, simple device, and many other advantages, it has become one of the most promising catalytic adsorbents for heavy metals removal.

The adsorption catalytic principle of nanomaterials mainly uses part of the cationic keys in the active free radicals such as the surface hydroxyl group and the environment to change its toxicity [66]. This affects the final migration and conversion of pollutants and achieves the fundamental purpose of treating pollutants in water. For example, the large π keys of nanomaterial graphite to polar organic pollutants interact to achieve polar adsorption. Another example is that combining graphite ole with magnetic material iron oxides can have a better adsorption and removal effect on heavy metal ions in water [64,66]. Furthermore, titanium dioxide nanomaterials can absorb organic pollutants through the interaction between superficial electrostatic effects and hydrogen bonds and can degrade their oxidation under ultraviolet exposure.

Carbon nano structural materials such as graphene generally have a uniform distribution, a large number of aperture distributions, and flat surface structures. As an ideal adsorbent, pollutant management research applications in the water field have achieved fixed results [16]. However, it is worth noting that the graphite sheet layer is prone to stacking due to the π–π interaction. This affects the effective exposure of the active electrical position hence failing to achieve the best adsorption catalytic effect [67]. On this basis, the scientific community has also developed oxide graphite and composite graphite through technologies such as oxidation–reduction, and their appearance has provided conditions for the universal application of nanomaterials.

(1) Graphene oxide (GO): The oxygen-containing functional groups (-OH, -C=O, -COOH, etc.) on the surface of the oxidized graphene make the adsorbent material have better hydrophilicity. In addition, it can be compatible with water when the heavy metal cations undergo a complex reaction to form complexes and exhibit excellent removal of pollutants in water. Ensafi Avval et al. [68] synthesized a new nanocomposite based on graphene oxide (GO). The ion adsorption of the binary mixture showed that the rich oxygen-containing groups on the surface of the nanocomposite played an important role in Pb^2+^. The order of ion adsorption affinity was Pb^2+^ > Cd^2+^ > Cr^2+^, and the adsorption and adsorption of ions on GO significantly depended on the pH value and had nothing to do with the ionic strength. Many studies have shown that the adsorption effect of oxidized graphene can be increased, mainly because the functional groups on the surface of the nanomaterials are complexed with heavy metal ions.

Oxide graphite is adsorbed with organic pollutants through the π–π key interaction, and the presence of oxygen-containing groups on the surface reinforces the adsorption of phenol organic matter. However, oxidized graphite can also be stranded in the water for a long time because of its excellent intimacy, and it becomes difficult to separate it with traditional separation methods. The emergence of composite graphite has solved this problem, expanding the broader platform for the adsorption of nanomaterials in the water environment.

**Figure 6:**
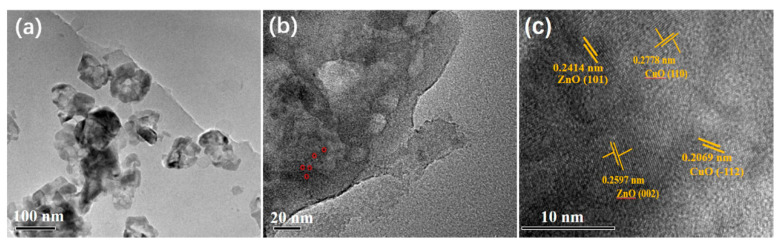
(**a**) low magnification; (**b**) high magnification; and (**c**) High Resolution (HR) TEM images of CZ-ESM nanocomposites. Reproduced [69] with permission from Elsevier, 2019.

(2) Composite graphite: Composite nanomaterials made been of graphite as a precursor system; in terms of the presence of reserved graphite, vinyl solves the problem of the dispersion of nanomaterials in water. In addition, in terms of new active materials, the introduction will give the catalytic material a higher adsorption catalyst, and the separation of the final water pollutant and the solid catalyst would be achieved. At present, more researches and applications in the field of water environmental pollution treatment are magnetic iron oxide graphite ole composite materials, that is, by using oxidized graphite and iron ions to adsorb heavy metal ions in water, after adsorption, the adsorbent is effectively recovered through magnetic separation technology. This reduces the burden of water environment purification. In the study of Kakavandi et al. [67], powder activated carbon (PAC) was magnetized by magnet ore nanoparticles (Fe_3_O_4_@C) as an adsorbent of lead ion (Pb^2+^) in aqueous solution. Pb^2+^ can reach the maximum single-layer adsorption capacity of 71 at 50 °C, 42 mg/g and Fe_3_O_4_@C. It has good potential for regeneration, and reusability and is easily regenerated by HCl solutions. There adsorption isotherm model parameters are shown in Table 3. It provides referenced adsorption models by using metal oxides/carbon. The adsorption process of such magnetic oxidation materials may be a promising heavy metal removal technology for use in large-scale and industrial applications.

(3) In addition to nano-carbon-based reductive adsorption catalysis, metal nanoparticles (e.g., Au, Ag, Cu, etc.) can also overcome kinetic obstacles. Xingsheng He et al. [69] synthesized CuO-ZnO (CZ) nanocomposites from biological waste—egg shell membrane (ESM)—using the biological template method. The results show:

①. Due to the effect of ultrasound, interconnected nanoparticles with irregular shapes are distributed on the film layer, and the average diameter of these nanoparticles is about 100 nm, as shown in Figure 6A.
②. As a result of the protein carbonization process, these nanoparticles (about 3–5 nm) are distributed on the surface of the film (carbonized ESM) (Figure 6B).
③. In addition, these small nanoparticles are identified as CuO or ZnO nanoparticles. The corresponding HR-TEM image in Figure 6C shows discontinuous lattice fringes. These results indicate that CZ-ESM is a heterogeneous nanocomposite material.

On this basis, using CZ-ESM nanocomposite as a catalyst at room temperature is a process of catalytic reduction of 4-NP to 4-AP.

**Figure 7:**
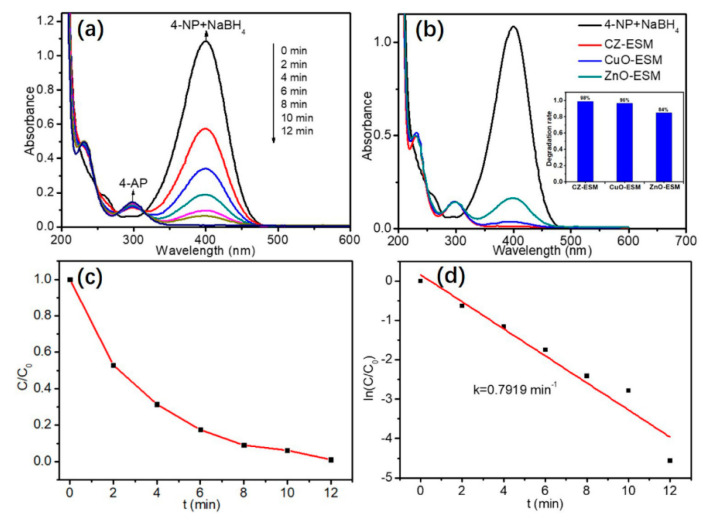
(**a**) Catalytic model of CZ-ESM nanocomposites for 4-NP reduction; (**b**) different samples for 4-nitrophenol at 12 min; and (**c**,**d**) plots of C/C_0_ and ln C/C_0_ versus reaction time (t) corresponding to the reduction of 4-NP catalyzed by CZ-ESM nanocomposites. Reproduced [69] with permission from Elsevier, 2019.

①. In Figure 7A, the formation of 4-AP is revealed. Compared with a single component, the CZ-ESM nanocomposite showed the best performance (Figure 7B). The degradation rate is calculated by the following formula: degradation rate (%) = (1 − C_t_/CO) × 100%, the maximum value is 98%.
②. Figure 6C shows the effective catalytic activity of the synthesized nanocomposite, which reduces to 4-AP after 12 min. The kinetic constant (k) is also calculated by using the pseudo-first-order kinetic model (dC/dt = kC). The relationship diagram in Figure 7D between ln(C/C_0_) and t confirms that the catalytic system follows the pseudo-first-order kinetic model, and the k value of CZ-ESM is 0.7919 min^−1^. The results show that the CZ-ESM nanocomposite shows significant activity and can be used as an effective catalyst for 4-NP reduction.

Besides, the composite materials assembled by the metal–organic frames (MOFs) and nanomaterials such as graphite are greatly integrated with the advantages of MOFs’ huge scale area, structural durability, and high gap rate with the high conductivity of graphite and stable structure without poison. Some of the shortcomings of a single component have shown an adsorption advantage stronger than traditional adsorption materials in the adsorption field. For example, in the preparation plan of Yan Zheng et al. [56], the addition of graphite vinyl materials enhanced the performance of composite materials in various ways. This indicated that graphite vinyl materials play an important role in composite materials. This is mainly due to the ion group and the aromatic sp^2^ domain. The graphite-based material can not only serve as a structural node but also participate in active cooperation in MOFs. Furthermore, the carboxylic acid roots and pyridines of graphical material enhance the positioning key and guide the growth of MOFs during assembly, thereby providing a more favorable structure. This shows that these special interactions are unique in materials based on MOFs/graphite. More importantly, the strategy brings the possibility of applying these composite materials to more fields than traditional materials, thereby achieving better results than traditional resources can achieve.

### 4.2. Existing Problems and Development of Nano-Sorption

The ideal adsorbent should have the characteristics of large adsorption capacity, good selectivity, a fast rate, a wide range of applications, strong circular performance, stable mechanical strength, no secondary pollution, and low preparation price [54,70]. Although the nanomaterials used and studied above can effectively expand the adsorption capacity and speed up the adsorption speed, due to the particularity of their structure, these nano-adsorbents have certain shortcomings and limitations [71]. Studies have also shown that it is difficult to design and the preferential selectivity to adsorb specific pollutants during the adsorption process is difficult to regulate. Therefore, a comprehensive and efficient adsorbent should possess the following characteristics: good adsorption thermodynamics, good kinetic and regenerative performance, and long service life [72]. The focus can be on the following two issues to inspire further studies to seek advancement in the research and development of nano-adsorbents.

(1) The nanomaterial adsorbents have a higher surface area than the general catalyst. It often reunites to form a secondary particle, which ultimately affects the adsorption process. To improve such conditions, Xiao Tang et al. [71] synthesized a titanium mine TiO_2_ nanometers with a single scattered large-scale two-dimensional nanostructure. Obvious quantum size effects were detected, and the light absorption capabilities were significantly enhanced. The light catalytic activity of TiO_2_ nanometers is catalyzed by light to restore Cr(VI) to Cr(III), which is manifested in the light of 200–800 nm. The reduction rate of hexavalent chromium reaches 99.8% in 15 min. At the same time, the original Cr(III) adulteration occurred spontaneously and triggered a significant visible-light-driven light catalytic effect. Under the light of 400–800 nm, it was restored within 100 min to produce 99% of Cr(VI). In this way, the huge scale area of the carrier material was used to fully and evenly spread the nanomaterial activity points. The high-altitude carrier also facilitates the transportation of the reactor. This measure solves the problems of the adsorption stability of nanomaterials.

(2) Most nanomaterial adsorbents are scattered in the water treatment environment in the form of powder. Due to the small particles, although heavy metals can be adsorbed efficiently, separation often encounters difficulties. To solve this restrictive problem. Yaru Yu et al. [37] prepared a series of polyvinyl alcohol (PVA)/graphene oxide (GO)-sodium alginate (SA) nanocomposite hydrogel beads through in-situ crosslinking to remove Pb^2+^. It was found that the adsorption capacity of the composite hydrogel. As the content of GO increases, the sphericity of the hydrogel beads decreases, the pore size increases, and a relatively loose network structure is formed, resulting in the permeability of the hydrogel and Pb^2+^. The adsorption capacity is improved, up to 279.43 mg g^−1^. In addition, the composite hydrogel has relatively good reusability for Pb^2+^ removal. Through the excavation and improvement of the above problems, it can be said that the nanomaterial adsorbent has achieved certain effects, but there are still limitations in application.

## 5. Hydrolysis Catalysis (Hydrogen Analysis Reaction and Oxygen Analysis Reaction)

The development of industrial technology in today’s human society still relies on the burning of fossil fuels, but fossil energy, as a non-renewable energy source, brings a series of problems such as serious secondary pollution, low product purity, and inability to recycle, which cannot be ignored. This, therefore, calls for the need for the implementation of the technology that ensures rapid economic development [1,73]. Hydrogen shows certain advantages as a clean and renewable energy source and is expected to replace fossil energy as a new energy supply source. The reason is that hydrogen is currently the fuel with the highest density (120 MJ/kg) among chemical fuels, and the product after combustion is still water that exists in nature. As a highly efficient recyclable energy material, it has been well studied by research and academics [16,50].

Before hydrogen energy is put into use and development, hydrogen preparation, storage, transportation, etc. are the key technologies [69]. To achieve efficient and large-scale preparation of hydrogen, hydrogen hydrogenation shows the characteristics of minimum pollution and the highest efficiency. Not only can it achieve long-term zero-emission hydrogen production, but its by-product oxygen can also be used for exhaust water exposure gas treatment, respiratory supply. This makes it an effective way to sustain sustainable production capacity.

However, the hydrolysis reaction is a very slow process. According to the standard Gibbs free energy change relationship, ΔG = +237.2 kJ mol^−1^ (Equation (6)). Therefore, additional energy must be provided to overcome the positive change in the freedom of Gibbs during the decomposition process [74]. At present, there are two main ways of energy source: (1) light catalytic hydrolysis, which is mainly limited by the width of the energy gap band mentioned above, and the absorption range of solar light is extremely narrow (mostly absorbing only 3–4% ultraviolet rays); and (2) electrocatalytic hydrolysis (OER) under the standard atmospheric pressure 1.23 V, and the theoretical electrode power of cathode hydrogen analysis reaction is 0.00 V. This indicates that the anode needs additional energy to make the hydrolysis response proceed smoothly [75]. These two points limit the ability of efficient hydropower dissolution processes to achieve hydrogen production and oxygen production. To achieve the goal of efficient catalysis, the restrictions imposed by the breakthrough of prohibited bandwidth and anode power are the main goals in the current catalytic field. Therefore, the development of efficient catalysts to promote the hydrolysis response process will be a practical and effective way to industrialize hydrogen energy.

H_2_O → H_2_ + 1/2 O_2_, ΔG = + 237.2 kJ mol^−1^ (6)

### 5.1. Photocatalysis

As the world energy crisis intensifies and pollution problems deteriorate, there is an urgent need to develop renewable energy and systems with high energy conversion efficiency to ensure sustainable human development [6]. As the most promising clean energy, solar energy has attracted widespread attention over the years [74]. In recent years, through the use of solar photocatalytic hydrogen production and oxygen production, there has been a substantial increase. The reason is that water can be used as a continuous photocatalytic resource, which will significantly reduce the possibility of secondary pollution. Moreover, light catalysis represents an effective way to transform solar energy into valuable chemical energy [54,76].

**Figure 8:**
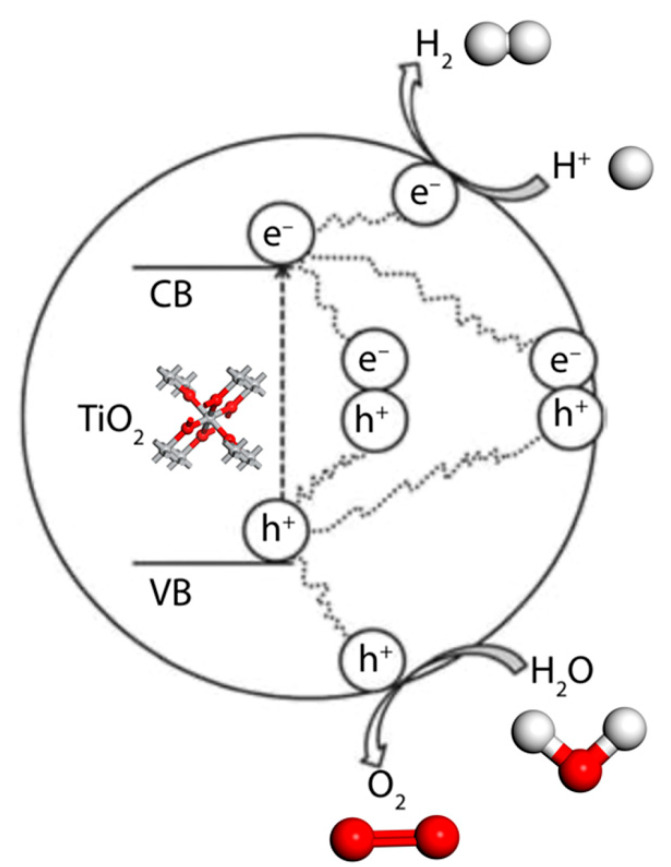
Schematic illustration of three basic processes of photogenerated electrons and holes during photocatalytic water splitting. Reproduced [74] with permission from Scrivener Publishing LLC, 2017.

Photocatalysis is achieved through the reduction of oxidation reaction on the surface of the light irradiating light catalyst, and the nanomaterials themselves do not participate in the reaction as light catalysts. For nanocatalysts, the quantum effect resulting from the smaller size makes it discontinuous, that is between the low-energy belt (VB) full of electrons and the high-energy belt (CB) without electrons. The size of the forbidden bandwidth determines the length of the absorbable light wave input, that is, the formula: into g (nm) = 1240/E_g_ (eV). There are three basic processes involved in photocatalytic hydrolysis, and Equations (7)–(9) show the detailed process in Figure 8. First, when the light catalyst is illuminated by a light greater than or equal to the forbidden bandwidth, the light electron and the light hollow h^+^ are generated in the valence band. Secondly, a large number of photovoltaic carriers will undergo a leapfrogging and will be transferred from the valence band to the guide belt, so that the guide belt has a reduced photoelectronic e^−^ while leaving an oxidizing light hole h^+^ in the valence band. Finally, there is no composite photovoltaic electron-empty point that migrates to the surface of the nanocatalyst, acts with the surface water of the catalyst, reduces H^+^ to H_2_, turns O^2-^oxidizes to O_2_, and completes the light catalytic reaction.

TiO_2_ + hν → hVB^+^ + eCB^−^ (7)

2H^+^ + eCB^−^ → H_2_ (8)

H_2_O + hVB^+^ → O_2_ (9)

In addition, as can be seen from the forbidden bandwidth and the light wave formula, the wider is the forbidden bandwidth of light catalytic materials, the shorter is the wavelength range of absorbable light. In this case, it only absorbs a very small proportion of ultraviolet light in solar light. The narrower is the bandwidth, the longer is the wavelength range of absorbable light, so that a larger part of the sunlight is used. It can be seen that minimizing the bandwidth of nanomaterials will help improve the light utilization rate. At the same time, even the photo-generated electrons and photo-generated holes that are separated by photo-generated carriers that respond to light may recombine and may be trapped by the surface defects of the nanomaterial. Therefore, extending the life of the electron–hole pair is to ensure effective means needed for catalytic efficiency.

Based on the above two points, in terms of evolution, the magnitude of the oxidative reduction capability of light catalyst depends on the energy-bearing structure of the selected light catalyst. If the electrical position at the top of the valence band is more positive, the oxidation capacity of the photovoltaic cavity is stronger with positive power generated by the interlocking of the electrons. However, if the electrical position at the bottom of the semiconductor belt becomes negative, the negative electronics separate from the photovoltaic cavity, and the reduction ability is stronger [17]. On this basis, thinking of the purpose of photocatalytic hydrogen production, it is necessary to make the bottom of the conductive belt of the photocatalyst more negative than the reduction of the electrical position of the water. In this way, the hydrogen ion in the water can be reduced to produce hydrogen. If the purpose is to correct the oxidation potential of the top of the valence band of the photocatalyst, it is possible to oxidize the oxide and lose two electrons to produce oxygen [55]. Therefore, to improve the efficiency of light catalysts as much as possible, under the conditions of ensuring PH, temperature, and light intensity, it is necessary to bring modifications. There are several main ways of carrying out the modifications:

(1) Pair can be retouched:

Surface composite deposition. Dengke Wang et al. [77] used a combination of simple and effective impregnation and light deposition methods. Common valent organic frame COFs were used as carriers and electronic suppliers, by depositing cadmium sulfide (CdS) nanoparticles on it to produce CdS-CTF-1 nano-composite material while maintaining the hydrogen production volume under the exposure of visible. The high activity of CdS-CTF-1 was mainly due to the small size effect of CdS nanoparticles, which exposed more points. Its excellent performance is also manifested in rapid electronic transmission rate and injection efficiency (K_ET_ = 0.18 × 10^9^ s^−1^, η_inj_ = 39.38%), 3–4 times faster than traditional nanomaterials.

Jiawei Liu et al. [6] mixed the pre-synthetic Au and Pt with GO through the wet chemistry synthesis method. Go was restored to rGO with NaBH_4_. The composite material obtained had good catalytic hydrogen analysis activity. This catalytic efficiency could exist under the conditions of visible light and near-infrared light, mainly due to the synergy of bimetal Au/Pt, the high-energy electrons induced by surface plasma resonance can be transferred from Auto Pt through the conductive rGO carrier, thereby enhancing the generation of hydrogen. In addition, large-scale contact between the gold and rGO has facilitated the transfer of more high-energy electrons.

Through characterization and microscopic research on precious metal composites, relevant research scholars [38] found that Au deposited in a single position in each Ag_2_S nanocrystal, and the core and growth of Pt and Os occurred in each Ag_2_S nanocrystal. In multiple positions, Ir is also the same sedimentation method, indicating the rich characteristics of Pt, Os, and Ir active position. Single slant crystals Ag_2_S have many small planes with different crystal spacing, and they provide a favorable position plane that matches precious metals so that precious metals can grow on and off the base. Using this point, with the increase of metal components, the combination of the nanoscale cross-section of the metal and the semiconductor can enable the photo bore carrier to efficiently perform oxidation–reduction reactions, so that the potential for catalytic applications will expand significantly.

(2) Improvement of crystal type

Ming Li et al. [65] developed the ethanol solvent thermal method to prepare a significant {001} surface (97%), and it significantly enhanced surface content of the ethanol solvent, which chemically adsorbed F^−^ for TiO_2_ nanometers. It had high stability with reduced surface energy, which resulted in a large percentage of {001} surfaces. This type of nanosheets adopts a clear morphology. When 1 wt% of Pt is loaded, the release rate of H_2_ is as high as 17.86 mmol h^−1^g^−1^, and the corresponding apparent quantum efficiency was determined to be 34.2%.

Hui Chen et al. [53] proved in their theory that the sharp titanium mine type TiO_2_-IrO_2_ solid sols have more active catalytic oxygenation response (OER) iridium catalytic activity levels than the standard OER catalyst IrO_2_. It is worth adding that the same situation has not been observed for similar products. This is because the sharp titanium mine TiO_2_ forbidden bandwidth is greater than the diamond type, and the conductive band is more negative. The result of this helps to separate the light electron reduction enhancement. The sharp titanium metal oxide also contains more defects and misalignments, which can produce more aerobic space to capture electrons that is not conducive to the implementation of light catalytic reactions. The sharp decline in the area of the golden stone table transformed from high-temperature sintering is the reason for the catalytic activity.

(3) Light sensitization

The photoactive dye, that is, the photosensitizer transfers its excess energy to the surface of the photocatalyst it covers to expand the excitation wavelength range of the catalyst to improve the utilization rate of long-wave radiation photons. This improves photon efficiency and enhances the photocatalytic performance. Stergiopoulos T. et al. [53] investigated the spectral properties and chemical adsorption properties of commercial organic ruthenium N_3_ and two new dyes Ru-CI and Ru-NCS, using these three organic ruthenium as sensitizers. Three kinds of nanobody TiO_2_ solar cells were assembled, and the photoelectric performance of the corresponding solar cells was studied. The results show that these three sensitizers have good absorption of visible light, and the absorption wavelength is extended to more than 700 nm. In the entire wavelength range of sunlight, the N_3_ sensitizing electrode has the largest light absorption intensity. At present, the application of photosensitizers is mainly to improve the photoelectric catalysis of fuel cells. The use of photosensitizers for water decomposition capacity is also promising based on the same mechanism. Photoactive compounds are adsorbed on the surface of photocatalysts, and the active material is used to stimulate the potential ratio. When the semiconductor conduction band potential is more negative, it is possible to transport photogenerated electrons to the conduction band of the semiconductor material. This has the advantage of expanding the excitation wavelength range and increasing the efficiency of the photocatalytic reaction.

### 5.2. Electric Catalysis

Since scientists discovered the phenomenon of electrolytic water in the 18th century, the scientific community has successively researched electrolytic water technology. The electrolyte is mainly composed of three parts: anode, cathode, and electrolyte [78]. When the electrolyte is water, hydrolysis can occur under the action of the current. The anode loses e-oxidation reaction oxygen production, and the cathode gets an e-reduction reaction to produce hydrogen [40]. Therefore, if you want to efficiently achieve the reduction of electrocatalytic oxidation, in addition to improving the transmission efficiency of electricity, optimizing the selection and construction of catalysts will be a more effective way to speed up the response process.

At present, the bottleneck regarding the application of electrocatalytic reduction in industrialization lies in how to optimize the performance of catalysts by regulating the composition of chemistry and physical structure. This is because the catalytic rate on the surface of the catalyst is mostly limited by its geometric nature and electronic structure, which affects the adsorption of intermediates, activism, and response energy base [79]. Currently, the most common optimization scheme is to use precious metal materials as the base of semiconductors, use their chemical stability, and provide a good interface for electrocatalysis. For example, some research scholars have used Ag_2_S as a precursor [80]. In addition, different precious metals can combine with epitaxial growth technology. The different crystal levels of the single-Italic crystal structure of the substrate Ag_2_S provides a favorable position for the matching of active components, as well as to design and derive composite materials. The electronic coupling effect can be used to improve electrochemical activity between different areas [38]. The use of this catalyst improves the response rate by effectively reducing the response base. Moreover, for some reactions, the effective use of the catalyst can change the course of the reaction and improve the efficiency of the response system, as indicated by Shumba et al. [20]. The composite material of multi-walled carbon nanotubes and gold nanorods was modified to modify the glassy carbon electrode, and the electrocatalytic behavior of the modified carbon electrode on the reduction of hydrogen peroxide was studied. They showed that the nanoparticles do not exist as nanoparticles alone or in the presence of multi-walled carbon nanotubes. The xylylene blue nano-dye can significantly improve the electron transfer kinetics. However, in terms of resource utilization, the scarcity of precious metal sources and high acquisition costs have seriously hindered its expansion and application in the field of electro-chemicals [74]. Therefore, how to stabilize the efficiency and ensure a relatively lower cost of catalysts production and regeneration is vital going forward.

**Figure 9:**
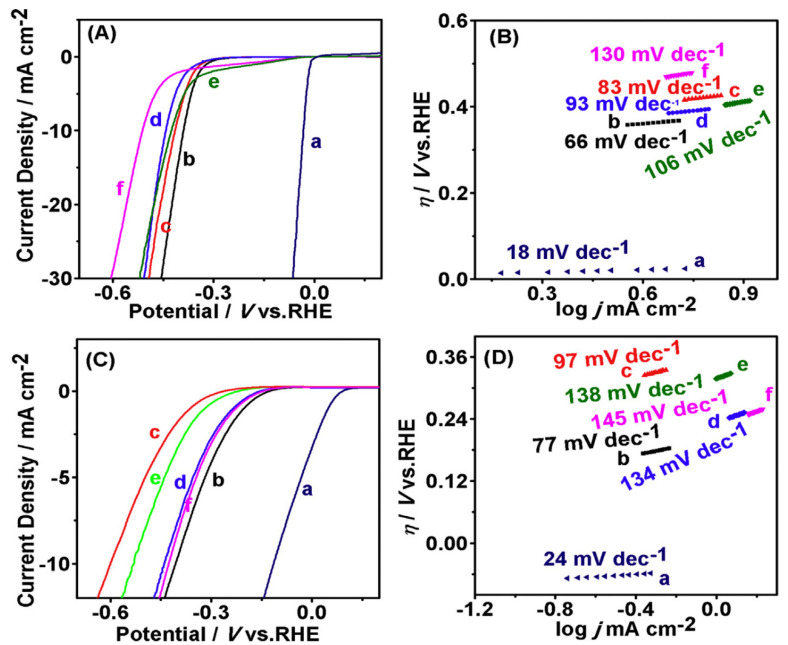
LSV curves of: (**b**) Fe_2_O_3_(1)-Co(1) NPs-N-GR/GCE; (**c**) Fe_2_O_3_(1)-Co(2) NPs-N-GR/GCE; (**d**) Fe_2_O_3_(2)-Co(1) NPs-N-GR/GCE; (**e**) Fe_2_O_3_ NPs-N-GR/GCE; (**f**) Co NPs-N-GR/GCE; and (**a**) Pt/C for HER measured at a scan rate of 2 mVs^−1^in 0.5 M H_2_SO_4_ (**A**) and in 1.0 M NaOH solution (**C**), and (**B**,**D**) the corresponding Tafel Plots. Reproduced [26] with permission from Elsevier, 2015.

The excellent HER catalytic performance of the bimetallic catalyst benefits from its unique composition and structural characteristics. Mingxuan Fu et al. [26] achieved the doping of N, the reduction of GO, and the formation of Fe_2_O_3_ NP and Co NP through a one-step calcination method. Through a series of simple, green, and cost-effective methods to successfully synthesize bimetallic upload nitrogen-doped graphene materials (Fe_2_O_3_-Co NPs-N-GR) in different proportions, to seek the best HER performance Fe_2_O_3_-CoNPs-N-GR material. The researchers used linear sweep voltammetry (LSV) to test the synthesized catalysts Fe_2_O_3_(1)-Co(1) NPs-N-GR, Fe_2_O_3_(1)-Co(2)NPs-N-GR, Fe_2_O_3_(2)-Co(1) NPs-N-GR, Fe_2_O_3_ NPs-N-GR, and Co NPs-N-GR 5 HER, as well as commercial catalysts Pt/C HER in 0.5 M H_2_SO_4_ solution (Figure 9A,B) and 1.0 M NaOH solution (Figure 9C,D). The results show that, among these catalysts, the NPs-N-GR catalyst has the best HER activity in 0.5 M H_2_SO_4_. The result is that the proper 1:1 ratio of iron and cobalt can improve the dispersibility of iron and enhance the catalytic activity of HER.

It is not difficult to find through characterization that Fe_2_O_3_(1)-Co(1)NPs-N-GR shows significant catalytic activity and excellent HER durability in a wide pH range. The electrocatalytic performance of Fe_2_O_3_(1)-Co(1)NPs N-GR for HER is better in acid solution (0.5M H_2_SO_4_) than in alkaline solution (1.0M NaOH). The overpotential is 0.36 V and the Tafel slope is 66 mV dec^−1^. The excellent HER catalytic performance of Fe_2_O_3_(1)-Co(1)NPs-N-GR benefits from its unique composition and structural characteristics, which are obtained by combining Fe_2_O_3_-Co nanoparticles and N co-doped graphene. This work provides new and valuable ideas for designing high-activity bimetallic N-doped graphene electrocatalysts for HER.

In addition to preparing electrocatalysts according to Sabatier’s principle, the researchers built a nanocomposite material to separate the adsorption surface from the desorption surface to increase the kinetic reaction rate [62]. According to studies, a simple strategy to synthesize the separation distance by a hydrothermal method is the ultra-thin molybdenum disulfide/nitrogen-doped. In addition, reduced graphene oxide nanocomposite of 9.5 A is conducive to the efficient hydrogen evolution reaction (HER) due to the synergy between the MoS_2_ nanosheets and the N-doped RGO film [81]. This type of material has extremely high hydrogen evolution activity under constant current conditions in an acidic environment and has good stability after 5000 cycles. This is because the adsorption of the metal active component on the surface of the hydroxyl group is very strong. As such, this type of catalyst has a higher hydrogen evolution activity than the traditional electrocatalyst under constant and high current conditions. The advantage of this strategy is that it overcomes the limitation of the Sabatier principle at a single active site and improves the catalytic activity from a kinetic perspective [33].

Although the electrolysis of water can be carried out in both acidic and alkaline environments, the OER reaction shows good performance under alkaline or neutral conditions, whereas the HER in alkaline environments shows 2–4 times slower than in acidic environments [82]. To achieve dual-function catalysis of both hydrogen and oxygen evolutions with a reaction rate of three orders of magnitude, the alkaline electrolytic cell can be put into industrial production. The selection and preparation of the hydrogen evolution catalyst in the alkaline medium are particularly critical. Ping Li et al. [20] used CoNiO_x_/reduced graphene oxide as a catalyst for electrolysis. It showed extremely low overpotential, rapid kinetics, and strong durability in long-term continuous electrolysis under the alkaline medium. What is more noteworthy is that, due to the in-situ electrocatalytic active substance (metal hydroxide) and oxygen defects, it can be further enhanced by the anode to improve its catalytic activity [57,67]. From this point of view, increasing the anode OER reaction in electrocatalysis can greatly improve the reaction rate, and the HER activity and reaction kinetics greatly depends on pH. The internal reason is that the activation energy of the reaction has a certain relationship with the enthalpy change. Water ionization of the kinetic energy barrier may also exert control over the overall reaction rate. The lower hydrolysis energy barrier requires that the catalyst have a sufficiently strong adsorption capacity for H^+^/OH^−^. Similarly, Chansheng Cao et al. [33] designed and prepared a self-supporting MOFs through the electrocatalytic method for high-efficiency electrochemical processes simply and economically. The characterization showed that the catalyst had a unique nanostructure and strong interaction between Ni and Fe active sites. The coupling effect made the OER reaction perform significantly well. It again had higher chemical stability than the general electrocatalytic materials, all of which benefited from the synergistic effect of the catalyst structure and composition.

Another new type of catalytic material is titanium dioxide nanomaterials is relatively abundant Earth resource and environmentally friendly with excellent catalytic hydrogen evolution strength. It also has outstanding oxygen evolution reaction efficiency [61]. Yiwei Hu et al. [78] prepared adjustable rutile anatase TiO_2_/reduced graphene oxide (RGO) with high OER activity. The study achieved a high current density of 10 mA/cm^2^ under an overpotential of 283 mV. This was possible due to the interaction between rutile and anatase (RGO and TiO_2_), which led to enhanced adsorption of hydroxyl groups. This adsorption was essential as it provided a favorable reaction environment for the surface and reduce activation energy, thereby granting the best OER performance. Going into the future, this technology will stimulate more designs that would improve the activity of OER, providing a new way of thinking for electrocatalytic hydrolysis.

## 6. The Current Status of Nano Research

Since the development of nanomaterials, it has become the most attractive research material due to its excellent performance in the field of catalysis, adsorption, and redox potential. In the fields of water research especially wastewater treatment and control and oxidative degradation, the research community has carried out multidimensional studies on nanomaterials. Other studies have also investigated the potential of changing the types of nanocarbon functional groups through redox in the field of catalysis. In the field of hydrogen and oxygen production by hydrolysis, the addition of external energy has provided power for the catalysis of nanomaterials, which has led to the development of low-energy and high-efficiency hydrolysis.

The achievements made by nanomaterials in just a dozen years are undeniable. However, in terms of science and technology, there are still some deficiencies in sustainable development research. In terms of mechanism research, good development has not been achieved [68]. The main reason is that in the aqueous environment among others, the relationship between the solid phase catalyst, the oxidant, and the target reactant is complicated, and, currently, no studies have been able to provide explicit information on the relationship between the three in the reaction process [83,84]. Under such circumstances, although the developed effective catalyst can achieve the conditions of high efficiency and low energy consumption, it is usually accompanied by disadvantages such as many components, high cost, and complicated preparation. At the same time, when the solid-phase nanocatalyst with a large specific surface area enters the water environment, it produces agglomeration and reduces the surface energy. This is often accompanied by a series of problems, such as loss of the active component of the metal [78], separation challenges, and other issues that significantly limit the use of nanomaterials. Based on this, it is of great significance to select composite catalysts to explore the basic law of its catalytic oxidation and to prove its reaction mechanism for the wider application of nanocatalysts, reducing the level of pollutants and increasing the energy conversion rate.

## 7. Existing Research Problems and Future Research Directions

The above nanomaterials are designed according to the specific characteristics of contaminated water bodies in Table 4. 

With the vigorous research and development of new materials, nanomaterials have advantages in water pollution control, such as the presence of surface effects, which increases their specific surface area [91,92]. In addition, large and enhanced surface activity and small size effects can effectively enhance their adsorption capacity [3,30,93]. The current research and development in the use of nanocatalysts provide prospects for the removal of pollutants in water and the utilization of renewable energy. The advantages, limitations, and characteristics of several basic advanced oxidation reactions are shown in Table 5. In this section, some issues worth exploring are highlighted and a future direction on nanocatalysts is also provided. The problem of water environment pollution and the development and utilization of water resources have important guiding significance [17,63].

(1) The flow of nanomaterials into the environment may have a profound impact on living things. Nanomaterials will inevitably flow into the environment during production, use, and disposal [20,21]. The activated or generated active oxygen clusters, such as the hydroxyl groups often mentioned above, will cause significant oxidative damage to biological cells. Dissolution will permanently exist in the environment and cause poisoning to organisms. To reduce the ecological and environmental risks promptly, it is necessary to improve the chemical stability of the nanomaterials, extend their cycle, or magnetically separate the metal nanomaterials to reduce the difficulties caused by filtration and centrifugal separation during the recovery process. On the other hand, completing the biosafety evaluation system should not be delayed. The use of uncontrollable nanomaterials for the long-term in the environment should be avoided [33].
(2) The limitation of the light absorption range of nanomaterials. At present, high-performance nanocatalysts generally have a wider bandgap and focus on the absorption of ultraviolet light, thereby greatly reducing the utilization rate of visible light and limiting large-scale industrial applications. Finding a more efficient and reliable light source is a way to improve the photocatalytic efficiency, such as amalgam lamps [59], diodes, etc., which is a problem that the research community needs to focus on to find efficient light sources.
(3) Development and utilization of other hydrogen storage energy. The development of low-cost hydrogen production capacity and efficient water electrolysis route is very attractive. However, it requires too much energy to overcome the activation energy of the anode side oxygen release reaction. Therefore, chemical hydrogen storage materials with high hydrogen storage capacity and easy dehydrogenation have also attracted people’s attention, such as hydrazine hydrate and ammonia boron [74], hydrocarbons (formic acid, methanol [70], etc.), and urea [75]. Such as non-toxic and flammable high-efficiency hydrogen storage materials, which can produce more hydrogen than water in a short time at room temperature. The development of catalysts that can promote the production of hydrogen from these hydrogen storage materials is the future development and utilization of water removal potential strategic goals for other hydrogen storage materials [6].
(4) Nanomaterials themselves are corroded in the environment, resulting in short service life. To achieve the long-term use of nanomaterials, the circulation of active ingredients flowing into the environment and the development of corrosion-resistant materials are particularly important development directives for regenerating nanomaterials [80].

## 8. Summary

Because of the remarkable achievements in science and technology and economic development, people have gradually paid attention to environmentally friendly sustainable energy technologies. Again, because of the deep danger of the depletion of the Earth’s energy, they have taken certain measures to prevent the situation from deteriorating and realize the common expectations of humanity. The development of a beautiful society has gradually become a universal desire in people’s hearts. To this end, this article summarizes the catalytic performance of nanomaterials in water pollution control and water resources utilization in recent years. This review focuses on the physical and chemical properties of nanomaterials and how they could achieve efficient energy use and maximum removal of pollutants.

Since the research of nanomaterials started, scientists have used different preparation methods to synthesize different shapes and sizes of nanomaterials and their composite materials. Through preparation adjustment and improved reaction conditions, the preparation conditions for preparing composite nanomaterials have been optimized. This has led to a new type of catalytic materials with the most stable structure and excellent performance. In general, when we combine nanomaterials for water and environmental pollution control to achieve efficient removal of pollutants in wastewater, the main degradation technologies used for different target pollutants are also different. Advanced oxidation is mainly aimed at organic pollutants and antibiotics, and adsorption catalysis mainly target heavy metal ions and organic pollutants in water. In terms of utilization of water resources capacity, as a renewable energy storage carrier, making hydrogen and oxygen as secondary energy to replace traditional fossil fuels to reduce greenhouse gas emissions is an effective means of future green energy and environmental protection. However, high-efficiency production capacity is also accompanied by problems such as increased costs and low utilization of light energy due to the use of electrical energy, leaving new research efforts for future nanocatalysis research to explore. At the same time, in the future, more efficient, low-cost, and stable nanocatalysts will require more research scholars to devote their efforts, which is also a development that not only meets the needs of contemporary people but also does not endanger the ability of future generations to meet their needs.

## Figures and Tables

**Table 1:** Standard electrode potentials of common oxidants [57,58].

Oxidant	Oxidation Potential (V) (Hydrogen Standard)
F_2_	3.03
•OH	2.80
SO_4_^−^•	2.60
•O	2.42
O_3_	2.07
S_2_O_8_^2−^	2.01
H_2_O_2_	1.77
Cl_2_	1.36
O_2_	0.40

**Table 2:** Average fiber diameters of NiO-ZnO_2_ systems before and after calcinations. Reproduced [60] with permission from Elsevier, 2014.

Sample Details	Average Fiber Diameters before Calcination (nm)	Average Fiber Diameters after Calcination (nm)
PVA (11 wt.%)	242 ± 35	Not applicable
PVA/NiO-ZrO_2_(90/10)	213 ± 44	30 ± 6
PVA/NiO-ZrO_2_(80/20)	195 ± 74	25 ± 9
PVA/NiO-ZrO_2_(50/50)	145 ± 30	106 ± 25

**Table 3:** Adsorption isotherm model parameters of Pb^2+^ adsorption onto Fe_3_O_4_@C at different temperatures. Reproduced [67] with permission from Wiley-VCH, 2015.

Isotherm Model	Temperature (°C)		
	20	35	50
Langmuir Isotherm			
q_m_ (mg/g)	58.82	62.5	71.42
K_L_ (L/mg)	0.068	0.07	0.06
R^2^	0.991	0.989	0.982
R_L_	0.04–0.22	0.045–0.23	0.052–0.025
Freundlich Isotherm			
K_F_ (mg/g (L/mg)${)}^{\frac{1}{n}}$	16	17.88	22.95
n	4.11	4.34	5.34
R^L^	0.959	0.958	0.931
Temkin Isotherm			
K_T_	2.6	3.73	16
B_1_	8.9	8.8	7.44
R^L^	0.928	0.916	0.864
Dubinin–Radushkevich			
q_m_ (mol/g)	5 × 10^−4^	4.8 × 10^−4^	4.5 × 10^−4^
D (mol^2^/KJ^2^)	0.0025	0.0022	0.0021
E (KJ/mol)	14.1	15	15.5
R^L^	0.953	0.944	0.898

**Table 4:** Water-related nanomaterials of water environment problems and treatment material.

Types	Nanomaterials	Performance	Conditions	References
Advanced Oxidation	TiO_2_	Degradation of methylene blue (MB)	25 min of sunlight	[85]
	TiO_2_	Degradation of methylene blue (MB)	pH = 7, UVC (95W, λ = 254 nm)	[17]
	TiO_2_	Catalytic reduction of Cr^5+^	Under 200–800 nm light irradiation	[71]
	Ti-Co@γAl_2_O_3_	Removal of total phenol and total organic carbon	pH = 8.2, ozone ventilation is 30 mg/min	[63]
	γ-Fe_2_O_3_@Co-MCM-41	Degradation of Orange II	Activation PMS	[86]
	MOFs-NiP	Degradation of Rhodamine B (RhB)	Activation PMS	[79]
	NiCo_2_O_4_	Degradation of Rhodamine B (RhB)	Neutral pH, current density 10 Ma·cm^−2^, activated PMS	[40]
	La_1.15_FeO_3_	Oxidative degradation of methyl orange	pH = 2.8–3	[62]
Catalytic Adsorption	Fe_2_O_3_@C	Adsorption of Pb^2+^ in water	pH = 6, Temperature is 20 °C, time is 60 min	[67]
	CuO-ZnO	Adsorption and catalytic reduction of 4-nitrophenol (4-NP) and strong antibacterial activity	(UV)λ = 498 nm	[69]
	GO	Adsorption of Pb^2+^, Cr^2+^, Cd^2+^ in water	pH = 5–6	[68]
	MOFs@GO (2:1)	Adsorption of methylene blue (MB) and uranium (U)	With the increase of temperature, the adsorption performance is enhanced	[56]
	Co_3_O_4_@rGO	Adsorption of Sb(III) and Sb(V)	pH = 9	[87]
	GO-Ag	Adsorption and removal of 2-nitroaniline (2-NA) and vat dyes (MG, MO, and EV)	pH = 9	[48]
	PVA/GO-SA	Adsorption of Pb^2+^ in water	When the GO content is 5 wt%	[17]
	PAL/PANI/AgNPs	Adsorption and catalytic reduction of Congo Red (CR), Adsorption of H_2_PO_4_	pH = 7.4	[83]
**Nanomaterials**		**Function**		**References**
TiO_2_ Nanosheets		Photocatalytic hydrogen production		[17,74]
CuOx-TiO_2_		Solar hydrolysis		[80]
Ni-Zn/TiO_2_ (9:1)		Photocatalytic hydrogen production		[88]
TiO_2_/WO_3_/Au/MWCNT		Photocatalytic hydrogen production		[82]
CdS-CTF-1		Photocatalytic hydrogen production		[77]
0D ZAIS CQD and 2D MoS_2_		Photocatalytic hydrogen production		[89]
MoS_2_/Graphene		Electrocatalytic oxygen production		[50]
CoNiO_x_/rGO		Electrocatalytic oxygen production		[20]
FeNi_2_Se_4_-NrGO		Bifunctional catalyst for OER and ORR		[80]
MoS_2_/N-RGO-180		Electrocatalytic oxygen production		[90]
Fe(Ni)-MOF		Electrocatalytic oxygen production		[33]
Manganese copper sulfide (MCS)		Photocatalytic and electrocatalytic hydrolysis		[39]
PRh/ZnO/Ni/Pt		Methanol oxidation		[68]

**Table 5:** The advantages, limitations, and characteristics of several basic advanced oxidation reactions.

Type of Oxidation Reaction	Advantages	Limitations	Features
Photooxidation	The reaction conditions are mild and can be carried out at room temperature and pressure Generally non-toxic and low price, no place restrictions. Low solar energy utilization.	The utilization rate of solar energy is low, and only less than 5% of ultraviolet light in sunlight can be used to excite photo-generated electrons and photo-generated holes	It mainly produces strong oxidizing free radicals under the irradiation of visible light (ultraviolet light), which has a good degradation effect on refractory organic wastewater
Electrooxidation	The oxidizers H_2_O_2_, O_3_, and ·OH have strong oxidizing properties and no secondary pollution Environmentally friendly, process controllable High processing efficiency	High-performance electrode materials need not only high hydrogen evolution potential and two-electron oxygen reduction reaction activity but also good stability and corrosion resistance	It regulates the generation of ·OH during the electrode reaction process to achieve the purpose of efficient oxidation. It is widely used in the treatment of organic pollutants and heavy metals
Fenton oxidation	Commonly used reagents are FeSO_4_·7H_2_O and H_2_O_2_ are non-toxic Mild reaction conditions and rapid response Can produce flocculation, simple operation, low investment	Affected by pH, it only works under acidic conditions The high temperature will make H_2_O_2_ The ratio of Fe^2+^ and H_2_O_2_ is strict The used Fenton reagent is difficult to separate and recycle	Under acidic conditions (pH = 2~5), it reacts to produce highly oxidizing ·OH, ·OH can degrade and mineralize most organic matter
Ozone oxidation	O_3_ is a strong oxidant, and its oxidation capacity is 1.5 times that of Cl_2_ O_3_ generates oxygen after oxidation, which is a green oxidant without secondary pollution Simple device, small footprint, easy to operate and control	Higher operating costs Strong oxidation selectivity, difficult to mineralize organic matter	It can be decomposed at room temperature to produce ·OH and monoatomic oxygen, which is effective in decolorization, deodorization, and deodorization of sewage, and removal of pollutants
